# Draft Genome Sequence of the Plant Pathogen *Streptomyces* sp. Strain 11-1-2

**DOI:** 10.1128/genomeA.00968-17

**Published:** 2017-09-14

**Authors:** Luke Bown, Dawn R. D. Bignell

**Affiliations:** Department of Biology, Memorial University of Newfoundland, St. John’s, Newfoundland and Labrador, Canada

## Abstract

*Streptomyces* sp. strain 11-1-2 is a Gram-positive filamentous bacterium that was isolated from a common scab lesion on a potato tuber. The strain is highly pathogenic to plants but does not produce the virulence-associated *Streptomyces* phytotoxin thaxtomin A. Here, we report the draft genome sequence of *Streptomyces* sp. 11-1-2.

## GENOME ANNOUNCEMENT

Members of the *Streptomyces* genus are Gram-positive filamentous spore-forming bacteria that are best known for their ability to produce a wide variety of secondary metabolites with useful biological activities (1). A small number of *Streptomyces* spp. can additionally form parasitic relationships with plants and cause economically important crop diseases, such as potato common scab (2). Most scab-causing streptomycetes produce thaxtomin A, a phytotoxic secondary metabolite that is essential for scab disease development (3). Recently, we isolated *Streptomyces* sp. strain 11-1-2 from a common scab lesion on a potato tuber harvested in Newfoundland, Canada (4). This strain was shown to be highly pathogenic to plants, and it produces at least one organic-soluble phytotoxin, but it does not produce thaxtomin A (4).

Genomic DNA was extracted from *Streptomyces* sp. 11-1-2 using the salting-out protocol (5) and was sequenced using the Pacific Biosciences RSII platform (Menlo Park, CA, USA) at the McGill University and Génome Québec Innovation Centre (Montréal, Québec, Canada). This produced a total of 198,563 raw subreads, with an average length of 11,877 bp, using two single-molecule real-time (SMRT) cells. The reads were assembled using the hierarchical genome assembly process (HGAP) (6) into two contigs totaling 11,655,206 bp, with an average coverage of 230× and an average G+C content of 70.85%. The analysis indicated that the genome consists of a single linear chromosome (11,603,877 bp) with a centrally located origin of replication, as well as a single plasmid (51,329 bp).

Gene prediction and annotation were carried out using the NCBI Prokaryotic Genome Annotation Pipeline version 4.2 (7). A total of 9,036 protein-coding genes, 64 tRNAs, and 18 rRNA genes (six 5S, six 16S, six 23S) were identified. Secondary metabolite biosynthetic gene clusters (BGCs) were predicted using antiSMASH version 4.0 (8), which identified 47 putative BGCs in the genome. This includes nine type I polyketide synthase (PKS) gene clusters, one type II PKS gene cluster, five nonribosomal peptide synthetase (NRPS) gene clusters, three hybrid PKS-NRPS gene clusters, and other BGCs for producing siderophores, lantipeptides, and terpenes. One BGC was found to be identical to the gene cluster known for producing the polyether ionophore nigericin (9). Also identified was a type I PKS BGC that is highly similar to the gene cluster that produces the phytotoxic ansamycin herbimycin in *Streptomyces hygroscopicus* (10).

Nucleotide BLAST analysis indicated that the *Streptomyces* sp. 11-1-2 chromosome sequence is most similar to that of *Streptomyces violaceusniger* Tü 4113 (96.8% nucleotide identity). It is currently unclear if the sequence differences between *Streptomyces* sp. 11-1-2 and *S. violaceusniger* exist at the strain level or the species level.

The *Streptomyces* sp. 11-1-2 genome sequence will be a valuable resource for characterizing the phytotoxic secondary metabolite(s) produced by this strain, as well as other virulence factors that contribute to the plant-pathogenic phenotype of the organism.

### Accession number(s).

The draft genome sequence of *Streptomyces* sp. 11-1-2 has been deposited in DDBJ/ENA/GenBank under the accession numbers CP022545 and CP022546. The versions described in this paper are the first versions, CPS022545.1 and CP022546.1, respectively.
